# Third-trimester estradiol is associated with isolated maternal hypothyroxinemia and attenuated TSH–FT4 coupling: evidence from rats and human astrocytes

**DOI:** 10.3389/fendo.2026.1795855

**Published:** 2026-04-30

**Authors:** Yajing Xu, Zitong Wang, Qingyan Cai, Honghong Duan, Huibin Huang

**Affiliations:** 1 Department of Endocrinology, The Second Affiliated Hospital of Fujian Medical University, Quanzhou, Fujian, China; 2 Independent Researcher, West New York, NJ, United States; 3 Department of Gynecology and Obstetrics, The Second Affiliated Hospital of Fujian Medical University, Quanzhou, Fujian, China

**Keywords:** DIO2, estradiol, isolated maternal hypothyroxinemia, OATP1C1, pituitary, pregnancy

## Abstract

**Introduction:**

Isolated maternal hypothyroxinemia (IMH), defined by low free thyroxine (FT4) with thyroid-stimulating hormone (TSH) within the reference range, is a clinically relevant thyroid phenotype in late pregnancy, when estradiol (E2) rises sharply. We tested whether third-trimester E2 is associated with IMH and whether complementary animal and cell data are compatible with a proposed model of pituitary adaptation.

**Methods:**

In 200 women at ≥28 gestational weeks (IMH, n = 100; euthyroid, n = 100), multivariable regression assessed associations of log2(E2) with IMH and FT4 after adjustment for gestational age and maternal age. Complementary experimental studies used gestational-stage Wistar rats, ovariectomized Wistar rats with 17β-E2 replacement, and Human Astrocytes treated with T4 ± E2 ± ICI 182,780.

**Results:**

Higher E2 was associated with IMH (OR 2.93 per doubling) and lower FT4 (−1.74 pmol/L per doubling), whereas the adjusted TSH–FT4 association was not statistically significant. In rats, late gestation and E2 replacement were associated with lower serum FT4, no statistically significant increase in TSH, lower pituitary T4, and higher pituitary Dio2 and Oatp1c1; in the OVX+E2 model, the pituitary T3/T4 ratio was also higher. In Human Astrocytes, E2 increased DIO2/OATP1C1 expression and the supernatant T3/T4 ratio, and ICI attenuated these effects, indicating estradiol responsiveness in a glial-like human cell system.

**Discussion:**

Together, these findings are compatible with altered local thyroid hormone handling under high-E2 conditions, but pituitary thyroid hormone metabolism was not directly assessed in the clinical cohort. The proposed model of pituitary adaptation should therefore be regarded as exploratory and hypothesis-generating rather than as a demonstrated human mechanism.

## 1 Introduction

Isolated maternal hypothyroxinemia (IMH) is characterized by low serum free thyroxine (FT4) with thyroid-stimulating hormone (TSH) within the reference range, typically in the absence of thyroid autoantibodies, and is a common thyroid phenotype during pregnancy (1). Its reported prevalence varies widely according to iodine status, gestational stage, and the reference interval applied (2–4). Whether IMH warrants levothyroxine (L-T4) replacement therapy and whether biochemical correction improves maternal or neonatal outcomes remain unsettled (1, 3, 5–8). Because L-T4 restores circulating FT4, the substrate for local T3 generation in maternal tissues and the maternal–placental–fetal unit, clarifying the pathophysiology of IMH remains clinically important (9).

Previous studies have suggested that iodine deficiency, environmental endocrine disruptors, obesity, iron deficiency, and imbalances in angiogenesis-related factors may contribute to IMH (3). However, these peripheral factors do not fully explain the core phenotype of IMH: declining FT4 without a compensatory increase in TSH. In the classic negative feedback regulation of the hypothalamic–pituitary–thyroid axis, reduced circulating thyroid hormone levels should stimulate TSH secretion (10). Therefore, IMH raises the possibility that pregnancy-related adaptations in hormone sensing or local thyroid hormone availability may involve pituitary-level processes.

Pituitary negative feedback is primarily mediated by intracellular triiodothyronine (T3) signaling (10, 11). In thyrotrophs, intracellular T3 is largely derived from local activation of thyroxine (T4) by type II deiodinase (DIO2) (12–14). In contrast, type III deiodinase (DIO3) mediates inactivation of T4 and T3 (15, 16), thereby regulating local bioactive T3 levels. Cellular entry of T4 and T3 also depends on specific transporters, including organic anion transporting polypeptide 1c1 (OATP1C1) (17) and monocarboxylate transporter 8 (MCT8) (10). Local T3 signaling suppresses transcription of the thyroid-stimulating hormone beta subunit (TSHβ) via thyroid hormone receptors; thus, TSHβ can serve as a molecular readout of pituitary feedback status, complementing circulating TSH as an indicator of pituitary output (14).

Estradiol (E2) rises progressively throughout pregnancy and reaches high concentrations in late gestation (18, 19). Clinical studies have reported a negative correlation between E2 and FT4 during pregnancy, suggesting that increasing E2 may parallel the decline in thyroxine (20, 21). In parallel, experimental data suggest that estrogen may influence pituitary thyroid hormone sensing and local T4-to-T3 activation (21–24). On this basis, we hypothesized that high-E2 conditions might be associated with altered local T4 uptake and T4-to-T3 conversion in experimental systems relevant to thyroid hormone handling and feedback. To examine this possibility, we combined a third-trimester clinical cohort analysis with gestational-stage and OVX+E2 rat models and *in vitro* experiments in Human Astrocytes. We evaluated DIO2/DIO3, OATP1C1, MCT8, and TSHβ/TSH in relation to local thyroid hormone availability to assess whether the multilevel findings were compatible with a proposed model of pituitary adaptation for the late-pregnancy phenotype of low FT4 with a relatively unchanged TSH response.

## 2 Materials and methods

### 2.1 Study subjects and clinical data

This retrospective study was approved by the Ethics Committee of the Second Affiliated Hospital of Fujian Medical University. Due to the retrospective nature of the study, the requirement for informed consent was waived. Clinical data of pregnant women who were hospitalized for delivery in the Department of Obstetrics and Gynecology of our hospital from June 2021 to August 2022 were reviewed. Ultimately, 200 pregnant women in late pregnancy (gestational age [GA] ≥ 28 weeks) were included and divided into the isolated maternal hypothyroxinemia group (IMH group, n = 100) and the euthyroid control group (EUT group, n = 100) based on their thyroid function in the third trimester.

Grouping and diagnostic criteria: The IMH group was operationally defined as FT4 < 12 pmol/L, with FT3 and TSH within the hospital laboratory reference ranges, together with negative thyroid autoantibodies (TGAb, TPOAb, and TRAb). The EUT group was defined as having FT3, FT4, and TSH within the same reference ranges, together with negative thyroid autoantibodies. The reference intervals used in this retrospective study (TSH 0.27–4.2 mIU/L, FT4 12–22 pmol/L, FT3 3.1–6.8 pmol/L) were the hospital laboratory intervals applied in routine clinical practice and were not pregnancy-specific trimester-derived reference intervals established from the study population. Accordingly, the IMH/EUT grouping in this study represents an operational classification for between-group comparison.

Inclusion criteria: (1) Singleton pregnancy, natural conception; (2) Normal thyroid function in the first and second trimesters (before 28 weeks); (3) Completion of thyroid hormone (FT3, FT4, TSH), thyroid autoantibody, and estradiol (E2) testing in the hospital during the third trimester; (4) No family history or past history of thyroid disease; (5) No L-T4 replacement therapy during pregnancy.

Exclusion criteria: (1) History of pituitary, thyroid, gonadal, or adrenal diseases; (2) Severe systemic diseases (e.g., malignancy, heart/respiratory failure, severe hepatic or renal dysfunction); (3) Twin or multiple pregnancies; (4) Known autoimmune thyroid disease or other documented autoimmune disorders that could affect thyroid function. GA was verified based on the last menstrual period combined with ultrasound dating. The retrospective dataset uniformly contained maternal age, GA, FT3, FT4, TSH, thyroid autoantibody results, and E2 measurements. TT4, TT3, and TBG were not included in the dataset used for the reported clinical analyses. Data on iodine status, iron status, body mass index, inflammatory markers, and medication exposure were not uniformly available and therefore were not included in the primary regression models.

### 2.2 Animal model construction and sampling

All animal experiments complied with the regulations of the Laboratory Animal Ethics Committee. Specific pathogen-free (SPF) healthy adult Wistar rats (females 6–8 weeks old, 220–260 g; males used for mating) were purchased from Shanghai SLAC Laboratory Animal Co., Ltd. Rats were housed in a standard SPF environment (12 h light/dark cycle, 22 ± 2 °C, humidity 50 ± 5%) with ad libitum access to food and water.

#### 2.2.1 Gestational stage rat model

Female and male rats were mated at a 1:1 ratio. The presence of a vaginal plug the next morning was designated as gestational day 0.5 (GD0.5). Gestational stages were set as non-pregnant (NP), GD6, GD12, and GD20 groups (n = 12 per group). At the corresponding time points, rats were anesthetized by intraperitoneal injection of sodium pentobarbital (1% w/v solution, 10 mg/mL) at 40 mg/kg body weight, after which blood was collected for serum isolation and pituitary tissues were rapidly harvested, snap-frozen in liquid nitrogen, and stored at −80 °C.

#### 2.2.2 Ovariectomy and E2 replacement model

Forty-five female Wistar rats were randomly divided into 5 groups (n = 9/group): Sham operation (Sham), OVX, OVX+vehicle (Veh), OVX+E2(0.7), and OVX+E2(2.1). Bilateral ovariectomy was performed under intraperitoneal sodium pentobarbital anesthesia (1% w/v solution, 10 mg/mL; 40 mg/kg body weight); in the Sham group, only periovarian fat was removed. Daily intraperitoneal injections began 3 days after surgery and continued for 21 days. Rats were weighed every other day to adjust the dosage. The OVX+E2(0.7) and OVX+E2(2.1) groups received 17β-E2 (Sigma-Aldrich; Cat# E2758) at doses of 0.7 (23) and 2.1 μg/100 g body weight, respectively, to generate two levels of estrogen replacement; the OVX+Veh group received an equivalent volume of corn oil. At the end of the intervention, serum and pituitary tissues were collected for subsequent assays.

### 2.3 Cell culture and intervention

Human Astrocytes (HA; ScienCell Research Laboratories, Carlsbad, CA, USA; Cat. No. 1800) were cultured in Astrocyte Medium (ScienCell; Cat. No. 1801) at 37 °C in 5% CO_2_. In this study, Human Astrocytes were used as a glial-like human cell system to examine E2-responsive changes in candidate thyroid hormone transport and activation pathways (15, 24–31), rather than as a direct surrogate for human pituitary regulation.

#### 2.3.1 Cell viability assay

Cells were seeded in 96-well plates and treated with different concentrations of 17β-E2 (0–50,000 pg/mL) or ICI 182,780 (5 × 10^−8 – 5 × 10^−5 mol/L; Sigma-Aldrich; Cat# I4409). After 48 hours, cell viability was detected using a CCK-8 kit (Meilun, Dalian; Cat# MA0218), and absorbance was read using a full-wavelength microplate reader (Multiskan Sky, Thermo Fisher Scientific). The control group received complete medium containing 0.01% DMSO.

#### 2.3.2 Intervention experiments

Four groups were established: T4, T4+E2, T4+ICI, and T4+E2+ICI. All groups were treated with T4 (20 pmol/L; Sigma-Aldrich; Cat# T2376). The final concentration of E2 was 15,000 pg/mL, and the final concentration of ICI was 5 × 10^−8 mol/L. The E2 concentration was selected from the dose-response and cell-viability experiments as the working concentration for the subsequent intervention experiments. After 48 h of intervention, cells were collected for mRNA and protein detection, and culture supernatants were collected for T3/T4 measurement.

### 2.4 Hormone and biochemical assays

#### 2.4.1 Serum and cell supernatant detection

Clinical serum FT3, FT4, TSH, and E2 were measured by electrochemiluminescence immunoassay (ECLIA) using a Cobas E601 analyzer (Roche Diagnostics) and matching reagents. Rat serum FT3, FT4, and E2, as well as T3 and T4 in cell culture supernatants, were also measured by ECLIA.

Rat serum TSH was detected by ELISA (Elabscience; Cat# E-EL-R0976), and the results are reported in the original assay units (ng/mL).

#### 2.4.2 Pituitary T3/T4 and TSH detection

Pituitary T3/T4: Pituitary tissues were homogenized in a protective buffer containing 20 mM Tris, 0.25 M sucrose, magnesium chloride, and DTT. Intracellular hormones were released using a previously described ethanol extraction procedure (32), with two repeated extractions using twice the volume of ethanol, and the supernatants were collected. The extracts were dried to remove solvent, reconstituted in PBS containing 0.5% BSA, and T3 and T4 were measured by ECLIA on the Cobas E601. These values were used for comparative between-group tissue readouts.

Pituitary TSH: Pituitary tissues were homogenized in the same protective buffer, centrifuged to obtain the supernatant, and assayed using the rat TSH ELISA kit used for serum (Elabscience; Cat# E-EL-R0976). Pituitary TSH results are reported in ng/mL.

### 2.5 RT-qPCR and Western blot

RT-qPCR: Total RNA was extracted using TRIzol Reagent (Invitrogen; Cat# 15596018CN), and cDNA was synthesized using the PrimeScript RT reagent Kit with gDNA Eraser (Takara Bio; Cat# RR047A). Amplification was performed using TB Green Premix Ex Taq II (Tli RNaseH Plus) (Takara Bio; Cat# RR820A) on a StepOne Plus Real-Time PCR System (Applied Biosystems). Glyceraldehyde-3-phosphate dehydrogenase (Gapdh/GAPDH) was used as the internal control, and relative expression was calculated using the 2^−ΔΔCt method. Primer sequences are listed in **Supplementary Table 10**.

Western blot: Total protein was extracted using RIPA lysis buffer (Beyotime; Cat# P0013B) and quantified with a BCA Protein Assay Kit (Solarbio; Cat# PC0020). Equal amounts of protein were separated by sodium dodecyl sulfate–polyacrylamide gel electrophoresis (SDS–PAGE) and transferred onto polyvinylidene difluoride (PVDF) membranes. Membranes were blocked with 5% (w/v) non-fat dry milk in Tris-buffered saline containing 0.1% Tween-20 (TBST) for 1 h at room temperature, incubated with the indicated primary antibodies overnight at 4 °C, and then with HRP-conjugated goat anti-rabbit IgG (H+L) secondary antibody (Affinity Bio; Cat# S0001) for 1 h at room temperature. Protein bands were visualized using an enhanced chemiluminescence (ECL) reagent (Thermo Scientific; Cat# 34579) and imaged with an ImageQuant LAS 4000 mini system (GE Healthcare). Detailed information for the antibodies used in Western blotting is provided in **Supplementary Table 11**.

### 2.6 Statistical analysis

Statistical analysis was performed using IBM SPSS Statistics 23.0 and GraphPad Prism 8.0. Continuous variables are presented as mean ± SD or median (interquartile range) according to distribution. Comparisons between two groups were performed using the independent-samples t test or Mann–Whitney U test. Multiple-group comparisons were performed using one-way ANOVA; Welch/Brown–Forsythe correction and appropriate *post hoc* comparisons were used when variances were unequal. All tests were two-tailed, and P < 0.05 was considered statistically significant. Because multiple experimental endpoints were evaluated in relatively small datasets, the animal and cell analyses were considered exploratory. Multiplicity was addressed within individual analyses through the specified *post hoc* procedures where applicable, but no single global multiplicity correction across all experimental endpoints was imposed; accordingly, the reported P values should be interpreted cautiously.

For ease of interpretation, E2 was log2-transformed in regression analyses so that effect estimates corresponded to a doubling of E2. In the clinical cohort, binary logistic regression was used to evaluate the association of log2(E2) with IMH status after adjustment for GA and maternal age. Multiple linear regression was performed with FT4 as a continuous outcome after adjustment for GA and maternal age; bias-corrected and accelerated bootstrap resampling (1,000 samples) was used to derive 95% confidence intervals. Sensitivity analyses and trend tests were performed using E2 quartiles. Exploratory analyses of TSH were performed after natural logarithm transformation [ln(TSH)] using models that included centered FT4, centered log2(E2), and their interaction; partial correlation analysis was used to assess the association between ln(TSH) and FT4 after controlling for GA and maternal age.

Indirect-effect analyses were performed using the PROCESS macro for SPSS (Model 4) with 5,000 bootstrap resamples (33). The clinical model was set as X = GA, M = log2(E2), and Y = FT4, with maternal age as a covariate; the animal model was set as X = gestational group, M = log2(E2), and Y = FT4. An indirect effect was considered significant when the bootstrap 95% confidence interval did not include zero. These indirect-effect analyses were exploratory and were intended to characterize associations within the specified models rather than to establish causal mediation.

In animal experiments, a General Linear Model (GLM) was used to evaluate the association between E2 and FT4 after adjustment for group. In cell experiments, two-way ANOVA was used to test the main effects and interaction of E2 and ICI, and one-way ANOVA with Tukey’s *post hoc* test was used for group comparisons.

## 3 Results

### 3.1 Clinical cohort: higher E2 is associated with IMH status and lower FT4, with a weak TSH–FT4 relationship

This study included 200 pregnant women in the third trimester (EUT, n = 100; IMH, n = 100). There were no significant differences in age and GA between the two groups. Compared with the EUT group, the IMH group had significantly higher serum E2 and significantly lower FT4, whereas no statistically significant differences were observed for FT3 or TSH (**Table 1**).

**Table 1:** Clinical and biochemical characteristics of EUT and IMH groups.

Variable	EUT (n = 100)	IMH (n = 100)	P value
Age (yr)	30.44 ± 3.767	30.77 ± 3.57	0.526
GA (wk)	38.43 (37.43, 39.57)	38.71 (37.71, 39.82)	0.395
E2 (pg/mL)	34285.72 ± 6119.01	37273.37 ± 8661.98	< 0.01
FT3 (pmol/L)	4.135 (4.00, 4.2575)	4.08 (3.7525, 4.30)	0.099
FT4 (pmol/L)	13.28 (12.8125, 13.6775)	10.455 (9.865, 11.065)	< 0.0001
TSH (mIU/L)	1.77 (1.48, 2.15)	1.80 (1.33, 2.11)	0.843

Data are presented as mean ± SD for normally distributed variables or median (interquartile range) for non-normally distributed variables. Comparisons between groups were performed using appropriate statistical tests. A two-sided P < 0.05 was considered statistically significant.

E2, estradiol; EUT, euthyroid controls; FT3, free triiodothyronine; FT4, free thyroxine; GA, gestational age; IMH, isolated maternal hypothyroxinemia; IQR, interquartile range; SD, standard deviation; TSH, thyroid-stimulating hormone.

**Figure 1:**
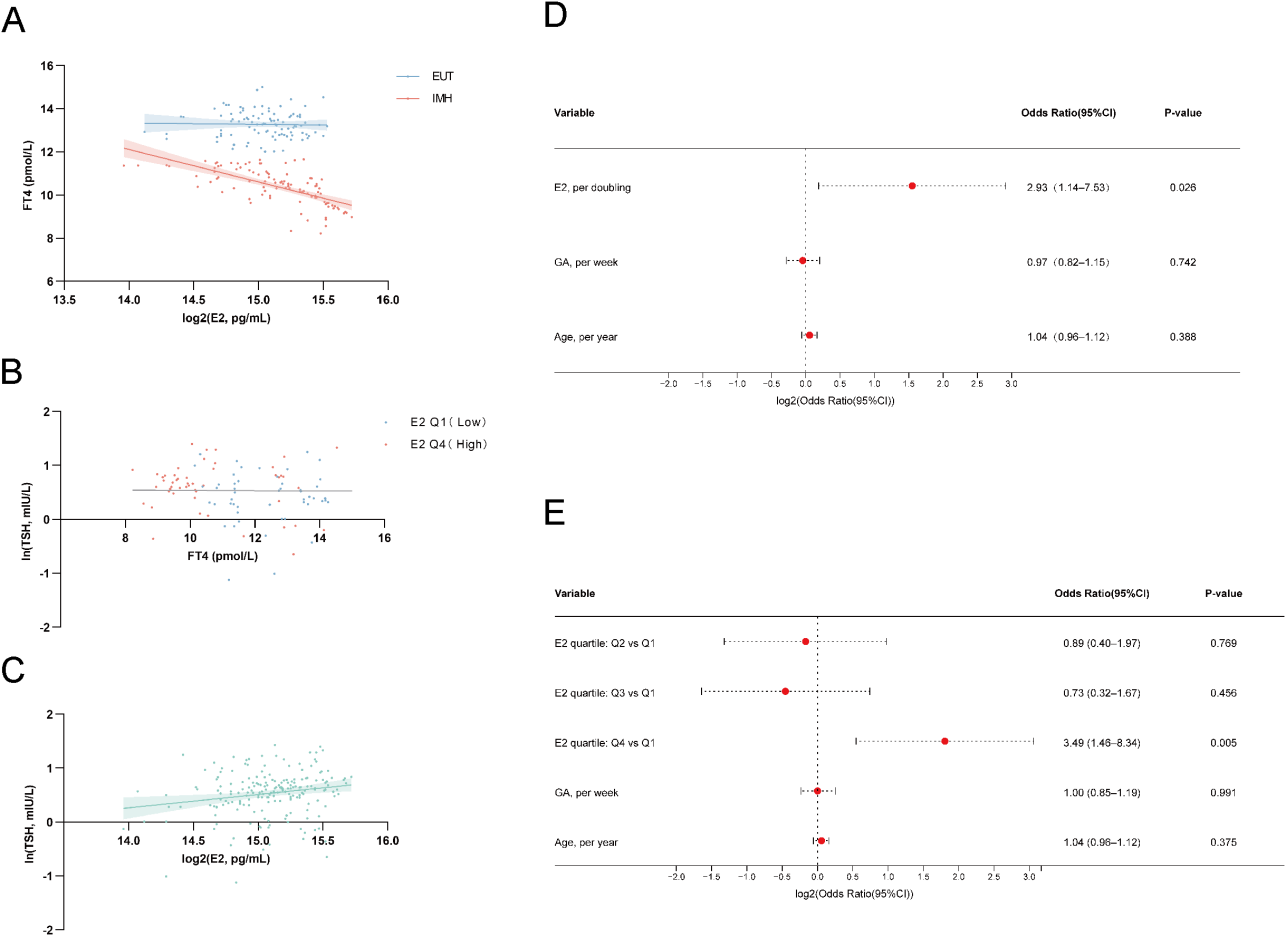
Associations of serum E2 with FT4, TSH, and IMH status in the clinical cohort. **(A)** Scatter plot illustrating the relationship between log2-transformed serum E2 concentrations and FT4 levels. Blue points and trend line represent the EUT group (n = 100); red points and trend line represent the IMH group (n = 100). Solid lines are unadjusted linear fits, and shaded areas indicate 95% CIs. **(B)** Relationship between serum FT4 and ln-transformed TSH levels, stratified by the lowest and highest E2 quartiles (Q1 vs. Q4). The gray solid line represents the unadjusted linear fit for the total study population (n = 200). The near-zero slope is consistent with a weak cross-sectional linear relationship between TSH and FT4. **(C)** Scatter plot depicting the relationship between ln(TSH) levels and log2-transformed serum E2 concentrations for the total population (n = 200). The solid line is the unadjusted linear fit with the shaded area indicating the 95% CI. **(D)** Forest plot showing ORs and 95% CIs from a multivariable binary logistic regression model evaluating the association between log2-transformed E2 and IMH status after adjustment for GA and maternal age. **(E)** Forest plot showing the ORs and 95% CIs from a multivariable binary logistic regression (Model B) evaluating the odds of IMH across E2 quartiles (Q2, Q3, and Q4 vs. Q1), adjusted for GA and maternal age. Higher odds of IMH were observed in the highest quartile (Q4 vs. Q1, P < 0.01). In Panels D and E, the x-axis is presented on a log2 scale. Statistical significance was determined by multivariable logistic regression. *P < 0.05; **P < 0.01; ***P < 0.001; ****P < 0.0001. CI, confidence interval; E2, estradiol; EUT, euthyroid controls; FT4, free thyroxine; GA, gestational age; IMH, isolated maternal hypothyroxinemia; ln, natural logarithm; log2, base-2 logarithm; n, number of subjects; OR, odds ratio; Q, quartile; Ref, reference; TSH, thyroid-stimulating hormone.

In binary logistic regression adjusted for GA and maternal age, log2(E2) showed a positive association with IMH status (per doubling of E2: OR = 2.925, P < 0.05; **Figure 1D**; **Table 2A**). When modeled by E2 quartiles, the highest quartile (Q4 vs Q1) showed increased odds of IMH (OR = 3.488, P < 0.01), with a significant trend across quartiles (P for trend < 0.05; **Table 2B**; **Figure 1E**); sensitivity analysis yielded consistent results (**Supplementary Table 1A**). Multiple linear regression further showed a negative association between log2(E2) and FT4 after adjustment for GA and maternal age (per doubling: B = −1.74 pmol/L, P < 0.01; **Table 2C**; **Figure 1A**). Indirect-effect analysis also suggested an indirect association of GA with FT4 through log2(E2) in the specified model (indirect effect = −0.0993, bootstrap 95% CI did not include 0; **Table 3**).

**Table 2:** Multivariable analysis of the association between E2, IMH status, and thyroid hormones.

Model and variable	OR/B	95% CI (or BCa 95% CI)	P value
A. IMH Status (Binary Logistic Regression) [1]			
log2(E2) (per 2-fold increase)	2.925	1.136, 7.534	< 0.05
GA (wk)	0.972	0.821, 1.151	0.742
Age (yr)	1.035	0.957, 1.118	0.388
B. IMH Status (E2 Quartiles as Predictor) [1]			
E2 Q1	Ref.	--	--
E2 Q2	0.887	0.399, 1.973	0.769
E2 Q3	0.73	0.320, 1.669	0.456
E2 Q4	3.488	1.460, 8.335	< 0.01
GA (wk)	1.001	0.846, 1.185	0.991
Age (yr)	1.037	0.957, 1.123	0.375
P for trend [2]	1.415	1.084, 1.848	< 0.05
C. FT4 Levels (Multiple Linear Regression) [3]	B (pmol/L)	BCa 95% CI	P (Bootstrap)
log2(E2) (per 2-fold increase)	-1.736	-2.686, -0.991	< 0.01
GA (wk)	0.028	-0.107, 0.151	0.624
Age (yr)	-0.039	-0.103, 0.024	0.224
D. ln(TSH) (Exploratory Linear Regression) [4]	B (coeff)	95% CI	P (Parametric)
GA (wk)	0.04	0.007, 0.073	< 0.05
Age (yr)	0.007	-0.009, 0.022	0.39
Centered FT4	0.02	-0.018, 0.059	0.302
Centered log2(E2)	0.205	0.011, 0.400	< 0.05 [5]
Interaction (Centered FT4 x Centered log2(E2))	-0.035	-0.157, 0.087	0.569

n = 200.

[1] Binary logistic regression adjusted for GA and maternal age. Model A: Nagelkerke R^2^ = 0.038, Hosmer-Lemeshow P = 0.049. Model B: Nagelkerke R^2^ = 0.111, Hosmer-Lemeshow P = 0.065.

[2] P for trend was calculated by entering E2 quartiles as an ordinal variable in the logistic model.

[3] Multiple linear regression with Bootstrap (BCa, 1000 resamples). VIF range: 1.01-1.12.

[4] Exploratory linear regression with centered variables and an interaction term. Reported coefficients, 95% CIs, and P values are from the parametric model. BCa bootstrap analysis (1,000 resamples) was used as a robustness check. Adjusted R^2^ = 0.052. VIF: 1.027-1.298.

[5] For centered log2(E2), the parametric model yielded P = 0.039, whereas the bootstrap analysis suggested only a trend (BCa 95% CI: -0.056 to 0.472; P = 0.087).

B, unstandardized coefficient; BCa, bias-corrected and accelerated; CI, confidence interval; E2, estradiol; FT4, free thyroxine; GA, gestational age; IMH, isolated maternal hypothyroxinemia; ln, natural logarithm; log2, base-2 logarithm; n, number of subjects; OR, odds ratio; Q, quartile; R^2^, coefficient of determination; Ref, reference; TSH, thyroid-stimulating hormone; VIF, Variance Inflation Factor.

**Table 3:** Indirect-effect analysis of the association between GA and FT4 through serum E2.

Path/Effect	Effect	SE/BootSE	t	P value	95% CI (LLCI, ULCI)	Bootstrap 95% CI
Path a: GA -> log2(E2) [1]	0.0572	0.0123	4.6495	< 0.001	0.0329, 0.0815	--
Path b: log2(E2) -> FT4 [2]	-1.7362	0.3507	-4.9507	< 0.001	-2.4278, -1.0446	--
Path c: Total Effect (GA -> FT4) [3]	-0.0716	0.0641	-1.1179	0.265	-0.1980, 0.0547	--
Path c’: Direct Effect (GA -> FT4) [4]	0.0277	0.0638	0.4341	0.665	-0.0981, 0.1535	--
Path a x b: Indirect Effect [5]	-0.0993	0.0393	--	--	--	-0.1870, -0.0345

n = 200. Analysis was performed using the PROCESS macro (Model 4), with maternal age included as a covariate.

[1] Path a represents the association between GA and the mediator (log2(E2)).

[2] Path b represents the association between the mediator and FT4, controlling for GA.

[3] Path c represents the total effect of GA on FT4.

[4] Path c’ represents the direct effect of GA on FT4 after controlling for the mediator.

[5] The indirect effect (a × b) was estimated using the Bootstrap method (5,000 resamples) and is considered significant as the Bootstrap 95% CI does not include zero.

BootSE, bootstrap standard error; CI, confidence interval; E2, estradiol; FT4, free thyroxine; GA, gestational age; LLCI, lower limit of confidence interval; log2, base-2 logarithm; n, number of subjects; SE, standard error; t, t-statistic; ULCI, upper limit of confidence interval.

The adjusted cross-sectional association between TSH and FT4 was not statistically significant. After adjustment for GA and maternal age, the partial correlation between ln(TSH) and FT4 was not statistically significant (r = 0.012, P = 0.865; **Supplementary Table 1B**; **Figure 1B**). In the exploratory ln(TSH) regression model, GA showed a positive association in the parametric model, but the corresponding bootstrap result did not confirm statistical significance. Centered log2(E2) also reached P < 0.05 in the parametric model, whereas the bootstrap analysis suggested only a trend (BCa 95% CI, -0.056 to 0.472; P = 0.087). Neither centered FT4 nor the interaction term was statistically significant (**Table 2D**; **Figure 1C**).

Based on these findings, we next examined related hormonal and pituitary readouts in animal models.

### 3.2 Gestational-stage rats: elevated E2 accompanies FT4 decline and pituitary Dio2/Oatp1c1 upregulation

**Figure 2:**
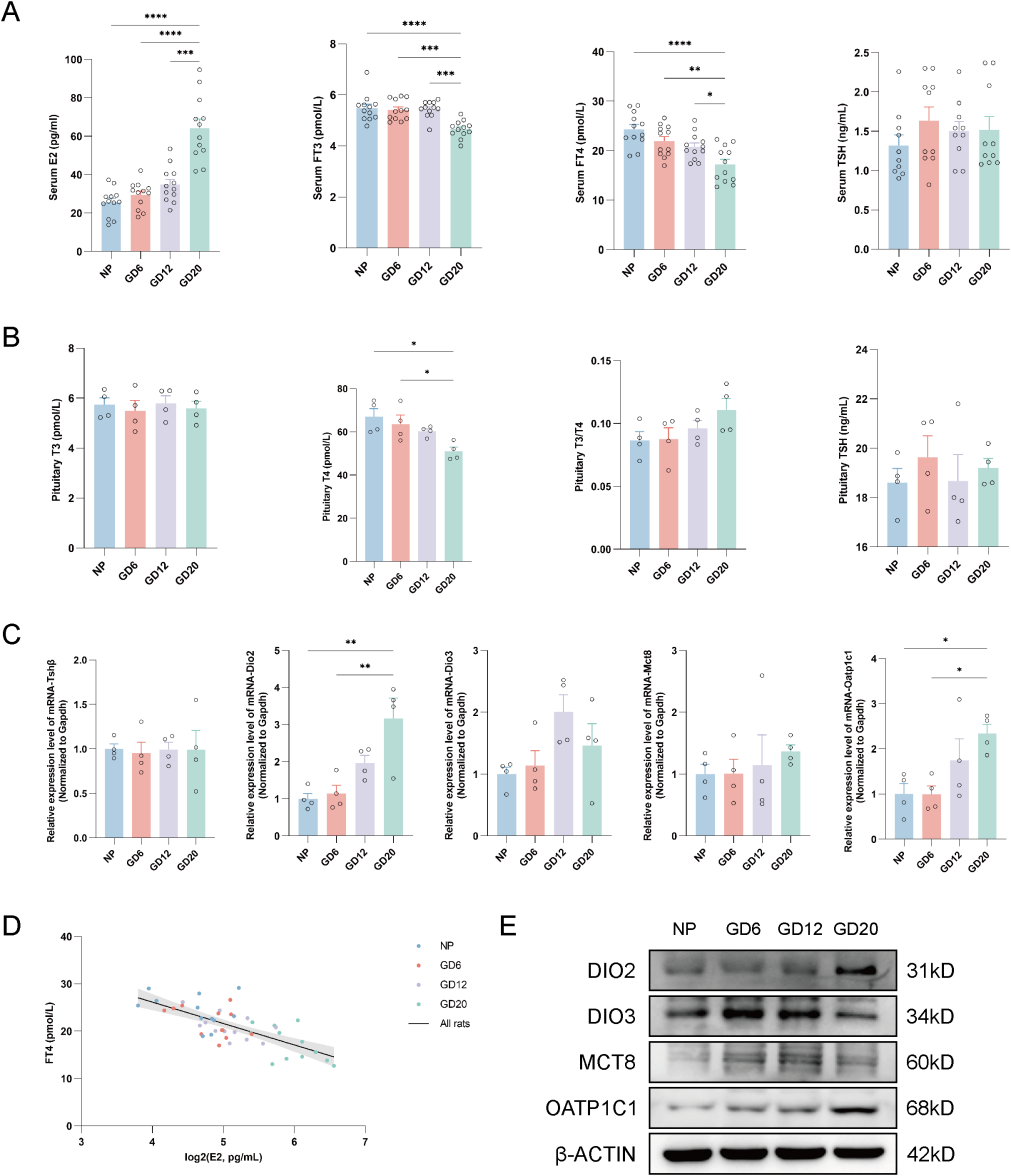
Pituitary–thyroid–estradiol axis alterations across gestational stages in rats. **(A)** Serum concentrations of E2, FT3, FT4, and TSH in NP and pregnant rats (E2/FT3/FT4: n = 12 per group; TSH: n = 10 per group). **(B)** Pituitary tissue levels of T3, T4, and TSH, and the T3/T4 ratio across gestational stages (n = 4 per group). **(C)** Relative mRNA expression of Tshβ, Dio2, Dio3, Mct8, and Oatp1c1 in the pituitary, normalized to Gapdh (n = 4 per group). **(D)** Scatter plot showing the relationship between serum FT4 and log2-transformed E2. The solid line is the unadjusted linear fit for the total population (n = 48). GLM analysis showed that log2(E2) was negatively associated with FT4 after adjustment for gestational group. **(E)** Representative Western blot images showing protein levels of DIO2, DIO3, MCT8, and OATP1C1 in the pituitary. β-ACTIN was used as the loading control. Data are shown as individual points with mean ± SE. n denotes independent biological replicates. Pituitary hormone values were obtained using an extraction-based assay and are presented as comparative tissue readouts. Differences among groups were assessed by one-way ANOVA or Welch/Brown–Forsythe-adjusted ANOVA followed by *post hoc* multiple-comparison tests. *P < 0.05, **P < 0.01, ***P < 0.001, ****P < 0.0001. ANOVA, analysis of variance; β-ACTIN, beta-actin; Dio2, type II deiodinase; Dio3, type III deiodinase; E2, estradiol; FT3, free triiodothyronine; FT4, free thyroxine; Gapdh, glyceraldehyde-3-phosphate dehydrogenase; GD, gestational day; GLM, General Linear Model; log2, base-2 logarithm; Mct8, monocarboxylate transporter 8; NP, non-pregnant; Oatp1c1, organic anion transporting polypeptide 1c1; SE, standard error; T3, triiodothyronine; T4, thyroxine; TSH, thyroid-stimulating hormone; Tshβ, thyrotropin subunit beta.

To examine the E2–FT4 association pattern in a physiological pregnancy context, we established a gestational-stage rat model from NP to GD20 (**Figure 2**). Serum E2 was significantly higher at GD20, whereas serum FT4 and FT3 were lower; no statistically significant difference in serum TSH was observed (**Figure 2A**; **Supplementary Table 2**). In pituitary tissue, T4 was lower at GD20 than at NP, whereas no statistically significant differences were observed for pituitary T3 or TSH (**Figure 2B**; **Supplementary Table 2**).

The pituitary T3/T4 ratio showed a numerical increase across gestation but did not reach statistical significance (NP: 0.087 ± 0.014; GD20: 0.111 ± 0.019; P = 0.18; **Figure 2B**; **Supplementary Table 2**); given the small number of pituitary samples, modest effects cannot be excluded. GLM analysis showed that log2(E2) remained negatively associated with FT4 after adjustment for gestational group (B = −3.74, P < 0.001; **Table 4A**; **Figure 2D**), whereas gestational group itself was not significant for FT4 (P = 0.50; **Table 4A**). Indirect-effect analysis suggested an indirect association of gestational stage with FT4 through log2(E2) in the specified model (indirect effect = −0.2308, bootstrap 95% CI did not include 0; **Table 5**). No statistically significant associations between ln(TSH) and E2 or FT4 were detected in the adjusted exploratory analyses (**Table 4B**; **Supplementary Tables 4**-**S5**), although limited power precludes exclusion of modest associations.

**Table 4:** GLM analysis of factors associated with serum FT4 and ln(TSH) levels in pregnant rats.

Model and predictor	F (df)	P value	Partial eta2	B (unstandardized)	95% CI for B
Panel A. Dependent variable: FT4 (n = 48) [1]					
Corrected Model	12.23 (4, 43)	< 0.001	0.53	--	--
GA Group	0.80 (3, 43)	0.5	0.05	--	--
log2(E2)	11.40 (1, 43)	< 0.001	0.21	-3.74	-5.97, -1.51
Panel B1. Dependent variable: ln(TSH)(n = 40) [2]					
Corrected Model	0.94 (4, 35)	0.45	0.1	--	--
GA Group	0.79 (3, 35)	0.51	0.06	--	--
log2(E2)	1.60 (1, 35)	0.21	0.04	0.16	-0.10, 0.41
Panel B2. Dependent variable: ln(TSH)(n = 40) [3]					
Corrected Model	1.12 (4, 35)	0.362	0.11	--	--
GA Group	0.51 (3, 35)	0.682	0.04	--	--
FT4	2.30 (1, 35)	0.138	0.06	-0.02	-0.06, 0.01

Analysis was performed using GLM (Type III Sum of Squares).

[1] Model A assessed the independent association of E2 with FT4 after adjustment for GA group (NP, GD6, GD12, GD20). Model fit: R^2^ = 0.532, Adjusted R^2^ = 0.489.

[2] Model B1 assessed the association between ln(TSH) and E2, adjusted for GA group. Model fit: R^2^ = 0.097.

[3] Model B2 assessed the association between ln(TSH) and FT4, adjusted for GA group. Model fit: R^2^ = 0.114.

B, unstandardized coefficient; CI, confidence interval; df, degrees of freedom; E2, estradiol; eta^2^, partial eta squared; F, F-statistic; FT4, free thyroxine; GA, gestational age; GD, gestational day; GLM, General Linear Model; ln, natural logarithm; log2, base-2 logarithm; n, number of subjects; NP, non-pregnant; R^2^, coefficient of determination; TSH, thyroid-stimulating hormone.

**Table 5:** Indirect-effect analysis of the association between GD and serum FT4 through E2 in rats.

Path/Effect	Effect	SE/BootSE	t	P value	95% CI (LLCI, ULCI)	Bootstrap 95% CI
Path a: GD -> log2(E2) [1]	0.0659	0.0081	8.1653	< 0.001	0.0497, 0.0822	--
Path b: log2(E2) -> FT4 [2]	-3.5019	1.0222	-3.4258	< 0.01	-5.5608, -1.4431	--
Path c: Total Effect (GD -> FT4) [3]	-0.3439	0.0622	-5.5329	< 0.001	-0.4690, -0.2188	--
Path c’: Direct Effect (GD -> FT4) [4]	-0.1131	0.0876	-1.2909	0.203	-0.2895, 0.0633	--
Path a x b: Indirect Effect [5]	-0.2308	0.0783	--	--	--	-0.3882, -0.0878

n = 48. Analysis was performed using the PROCESS macro (Model 4).

[1] Path a represents the association between GD and the mediator (log2(E2)).

[2] Path b represents the association between the mediator and FT4, controlling for GD.

[3] Path c represents the total effect of GD on FT4.

[4] Path c’ represents the direct effect of GD on FT4 after controlling for the mediator.

[5] The indirect effect (a × b) was estimated using the Bootstrap method (5,000 resamples) and is considered significant as the Bootstrap 95% CI does not include zero.

BootSE, bootstrap standard error; CI, confidence interval; E2, estradiol; FT4, free thyroxine; GD, gestational day; LLCI, lower limit of confidence interval; log2, base-2 logarithm; n, number of subjects; SE, standard error; t, t-statistic; ULCI, upper limit of confidence interval.

At the molecular level, pituitary Dio2 and Oatp1c1 mRNA levels were significantly higher at GD20 than at NP and GD6 (**Figure 2C**; **Supplementary Table 3**), and representative Western blots suggested increased DIO2 and OATP1C1 protein signals at GD20 (**Figure 2E**).

### 3.3 OVX+E2 replacement: reduced FT4 without a statistically significant increase in TSH, accompanied by a higher pituitary T3/T4 ratio and higher Dio2/Oatp1c1 mRNA

**Figure 3:**
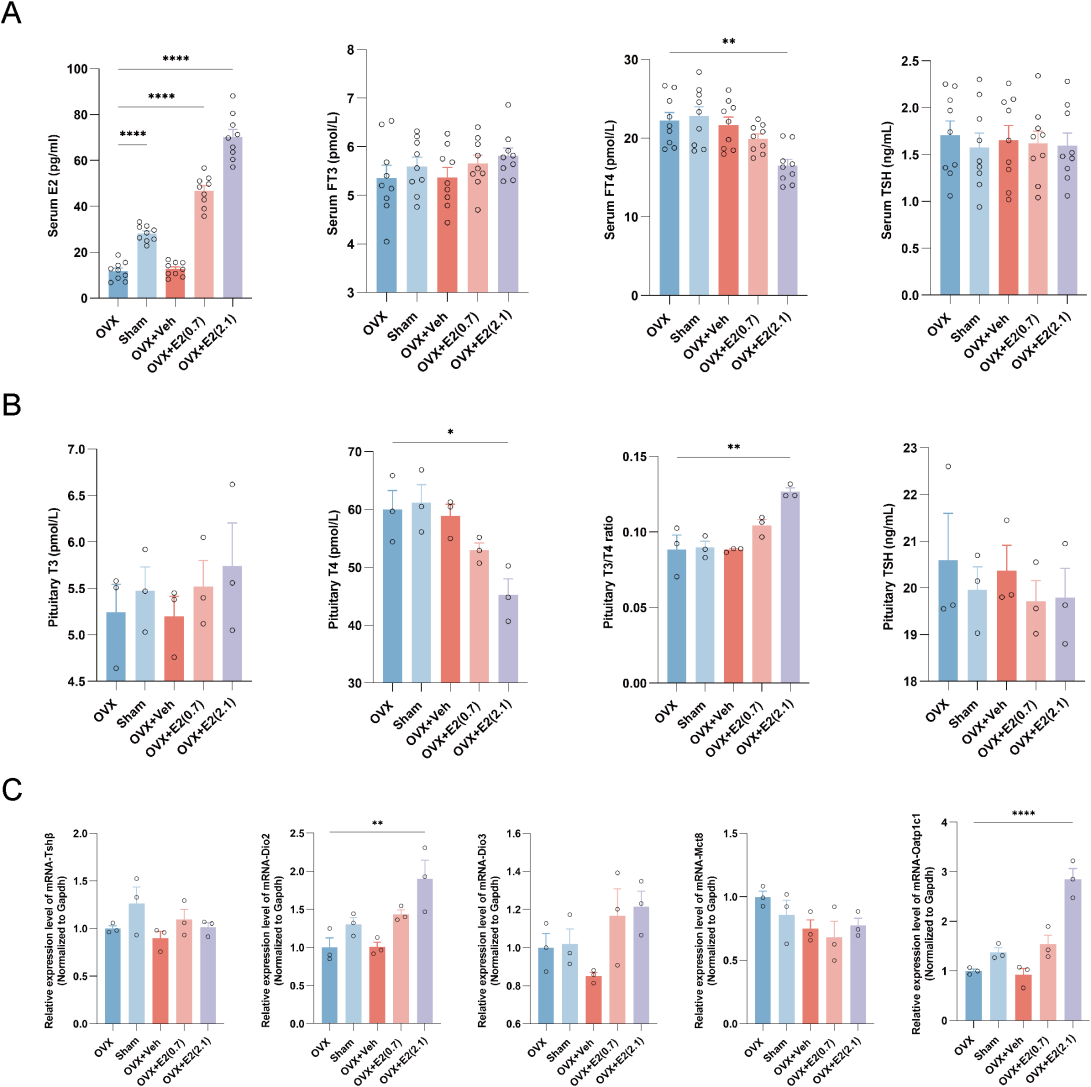
Impact of E2 replacement on the thyroid-pituitary axis in an OVX rat model. **(A)** Serum levels of E2, FT3, FT4, and TSH across five groups: Sham, OVX, and OVX rats treated with Veh or different doses of E2 [OVX+E2(0.7) and OVX+E2(2.1)] (n = 9 per group). **(B)** Pituitary tissue T3, T4, T3/T4 ratio, and TSH across the five groups (n = 3 per group). **(C)** Relative mRNA expression of Tshβ, Dio2, Dio3, Mct8, and Oatp1c1 in the pituitary, normalized to Gapdh (n = 3 per group). Data are shown as individual points with mean ± SE. n denotes independent biological replicates. Pituitary hormone values were obtained using an extraction-based assay and are presented as comparative tissue readouts. Statistical significance was determined by one-way ANOVA or Welch/Brown–Forsythe-adjusted ANOVA when variances were unequal, followed by *post hoc* multiple-comparison tests. Comparisons were performed using the OVX group as the baseline reference unless otherwise indicated. *P < 0.05; **P < 0.01; ***P < 0.001; ****P < 0.0001 vs. the OVX group. ANOVA, analysis of variance; Dio2, type II deiodinase; Dio3, type III deiodinase; E2, estradiol; FT3, free triiodothyronine; FT4, free thyroxine; Gapdh, glyceraldehyde-3-phosphate dehydrogenase; Mct8, monocarboxylate transporter 8; Oatp1c1, organic anion transporting polypeptide 1c1; OVX, ovariectomized; SE, standard error; Sham, sham-operated; T3, triiodothyronine; T4, thyroxine; TSH, thyroid-stimulating hormone; Tshβ, thyrotropin subunit beta; Veh, vehicle control.

In the OVX model, E2 supplementation resulted in a dose-dependent increase in serum E2 (**Figure 3A**; **Supplementary Table 6**). Serum FT4 was significantly decreased in the OVX+E2(2.1) group compared with the OVX and OVX+Veh groups, whereas no statistically significant differences were observed for FT3 or TSH (**Figure 3A**; **Supplementary Table 6**). In the OVX+E2 model, reduced FT4 occurred without a statistically significant between-group difference in FT3 or TSH. At the pituitary level, T4 decreased and the T3/T4 ratio increased in the OVX+E2(2.1) group, whereas no statistically significant differences were observed for pituitary T3 or TSH (**Figure 3B**; **Supplementary Table 6**). Pituitary mRNA analysis showed significant upregulation of Dio2 and Oatp1c1 in the OVX+E2(2.1) group, whereas no statistically significant differences were observed for Tshβ, Dio3, or Mct8; because these comparisons were based on small subgroup samples, non-significant findings should be interpreted cautiously, and modest effects cannot be excluded (**Figure 3C**; **Supplementary Table 7**).

### 3.4 Human astrocytes: E2 upregulates DIO2/OATP1C1 and increases the supernatant T3/T4 ratio, reversible by ICI

**Figure 4:**
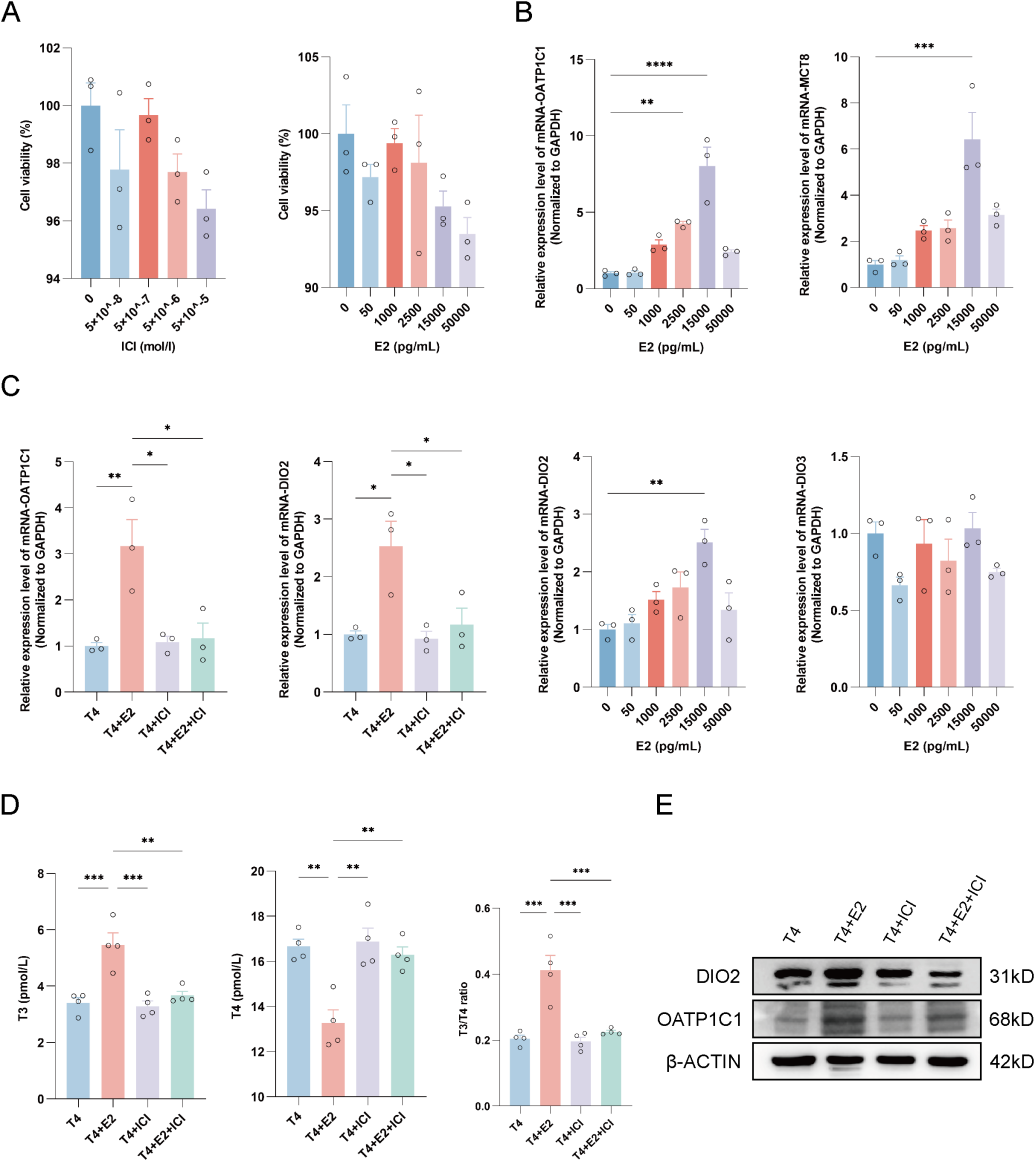
E2-responsive changes in candidate thyroid hormone transport and activation pathways in Human Astrocytes. **(A)** Cell viability of Human Astrocytes treated with varying concentrations of ICI or E2 for 48 h (n = 3). **(B)** Dose-response effects of E2 on the relative mRNA expression of DIO2, DIO3, OATP1C1, and MCT8 (n = 3). **(C)** Relative mRNA expression of OATP1C1 and DIO2 after treatment with T4 alone or in combination with E2 and/or ICI (n = 3). **(D)** Concentrations of T3 and T4, and the T3/T4 ratio in culture supernatants after treatment (n = 4). **(E)** Representative Western blot images of DIO2 and OATP1C1 under the same treatment conditions as in Panel **(C)**. β-ACTIN was used as the loading control. Data are shown as individual points with mean ± SE in Panels A–D. Relative mRNA expression was normalized to GAPDH. n denotes independent biological replicates. Based on the dose-response and cell-viability experiments, 15,000 pg/mL E2 was used as the working concentration for the intervention experiments. Panels A–C were analyzed by one-way ANOVA or Welch/Brown–Forsythe-adjusted ANOVA followed by *post hoc* multiple-comparison tests. Panel D is summarized quantitatively in **Table 6**; one-way ANOVA with Tukey’s *post hoc* test was used for overall comparison, and two-way ANOVA was used to evaluate E2 × ICI interactions. Panel E is representative. *P < 0.05; **P < 0.01; ***P < 0.001; ****P < 0.0001. ANOVA, analysis of variance; β-ACTIN, beta-actin; DIO2, type II deiodinase; DIO3, type III deiodinase; E2, estradiol; GAPDH, glyceraldehyde-3-phosphate dehydrogenase; ICI, ICI 182,780; MCT8, monocarboxylate transporter 8; OATP1C1, organic anion transporting polypeptide 1c1; SE, standard error; T3, triiodothyronine; T4, thyroxine.

CCK-8 assays showed no significant difference in cell viability after 48 h of E2 or ICI treatment compared with controls (**Figure 4A**). Dose-response experiments revealed significant overall differences in DIO2 and OATP1C1 mRNA across E2 concentrations (overall P < 0.01 for both), whereas DIO3 showed no significant overall difference (overall P = 0.141) (**Figure 4B**; **Supplementary Table 8**). Based on the dose-response and viability findings, 15,000 pg/mL E2 was selected for the subsequent intervention experiments (**Figure 4B**).

Compared with the T4 baseline, E2 increased DIO2 and OATP1C1 mRNA, and this upregulation was attenuated by ICI (n = 3; **Figure 4C**; **Supplementary Table 9**). Representative Western blots suggested the same directional pattern for DIO2 and OATP1C1 protein signals (**Figure 4E**). Analysis of culture supernatants (n = 4) showed that E2 increased T3, decreased T4, and elevated the T3/T4 ratio (**Table 6**; **Figure 4D**). ICI attenuated these E2-induced changes, and a significant E2 × ICI interaction was observed (**Table 6**).

**Table 6:** Effects of E2 and ICI on supernatant thyroid hormone levels in human astrocytes.

Parameter (unit)	T4	T4+E2	T4+ICI	T4+E2+ICI	One-way P	Interaction P
T3 (pmol/L)	3.41 ± 0.37 (a)	5.47 ± 0.85 (b)	3.29 ± 0.37 (a)	3.67 ± 0.30 (a)	< 0.001	< 0.001
T4 (pmol/L)	16.66 ± 0.65 (a)	13.27 ± 1.17 (b)	16.87 ± 1.23 (a)	16.29 ± 0.70 (a)	< 0.001	< 0.05
T3/T4 ratio	0.20 ± 0.02 (a)	0.41 ± 0.09 (b)	0.20 ± 0.03 (a)	0.22 ± 0.01 (a)	< 0.0001	< 0.01

Data are presented as mean ± SD (n = 4 per group). n denotes independent biological replicates. One-way ANOVA was used for overall comparison, followed by Tukey’s *post hoc* test; two-way ANOVA was used to evaluate E2 × ICI interactions. Within each row, means followed by different superscript letters (a, b) indicate a statistically significant difference (P < 0.05). When the E2 × ICI interaction was significant, simple-effects comparisons were performed with Šidák correction.

ANOVA, analysis of variance; E2, estradiol; ICI, ICI 182,780; SD, standard deviation; T3, triiodothyronine; T4, thyroxine.

## 4 Discussion

The present study combines a retrospective clinical analysis with animal and cell-based experiments to examine whether E2-associated changes in local thyroid hormone handling could help explain the late-pregnancy pattern of lower FT4 with relatively unchanged TSH. In this framework, high-E2 conditions may be associated with enhanced local T4 uptake and T4-to-T3 activation via DIO2 and OATP1C1, potentially buffering pituitary T3-related feedback despite lower peripheral FT4. Because pituitary thyroid hormone metabolism was not directly assessed in the clinical cohort, this proposed mechanism remains exploratory and hypothesis-generating.

In the clinical cohort, third-trimester E2 was associated with IMH status and lower FT4 after adjustment for GA and maternal age, whereas the adjusted cross-sectional association between TSH and FT4 was not statistically significant. These observations indicate that low-FT4 states can coexist with TSH values within the reference range in this cohort, but they do not establish the clinical utility of FT4 relative to TSH. Because late pregnancy is accompanied by estrogen-related changes in thyroid hormone binding, including higher TBG, and FT4 measured by routine immunoassay may be method-dependent in this setting, the observed inverse E2–FT4 association in the clinical cohort should be interpreted cautiously (34–36). The indirect-effect analyses were exploratory and should not be interpreted causally.

Residual confounding remains an important source of uncertainty. Iodine deficiency or iron deficiency could lower FT4 independently of E2 and thereby exaggerate an inverse E2–FT4 association if unevenly distributed across the exposure range. Body mass index or adiposity may influence both estrogen exposure and thyroid indices, potentially attenuating or accentuating the observed association. Inflammatory activity and medication exposure could also influence thyroid hormone concentrations, binding, transport, or feedback-related readouts independently of E2. Because these factors were not uniformly measured, they may have influenced both the direction and magnitude of the reported association.

Within the experimental systems, the physiological gestational-stage model and the OVX+E2 supplementation model showed several features resembling the peripheral hormone pattern observed clinically, most notably lower FT4 in the setting of no statistically significant TSH increase. The two animal models should nevertheless be regarded as complementary rather than interchangeable. In the clinical cohort, lower FT4 was observed without a statistically significant difference in FT3, whereas in the gestational-stage rat model both FT4 and FT3 decreased at late gestation. By contrast, the OVX+E2 model more closely resembled the human peripheral hormone pattern in showing reduced FT4 without statistically significant changes in FT3 or TSH. Because some of these comparisons were based on small subgroup samples, modest effects cannot be excluded.

This cross-system discordance in circulating FT3 likely reflects species-specific thyroid hormone handling during pregnancy, differences in circulating hormone-binding physiology, and the distinction between a retrospective late-pregnancy clinical grouping and controlled experimental models. Across the experimental systems, however, the more consistent signal was lower pituitary T4 together with relatively stable pituitary T3 and TSH readouts and higher Dio2/Oatp1c1-related signals, findings that are compatible with altered local thyroid hormone handling in these systems and with prior experimental literature (21–24, 37, 38), but do not establish a validated human pituitary mechanism. The pituitary T3/T4 measurements should also be viewed as comparative tissue readouts derived from an extraction-based protocol rather than as independently matrix-validated absolute pituitary hormone concentrations.

Following function-oriented screening of candidate deiodinases and transporters, the most consistent directional changes under high-E2 conditions involved Dio2 and Oatp1c1, which were therefore examined further in a controlled glial-like system. Tshβ was retained as an exploratory transcriptional readout related to feedback-associated signaling, but not as direct evidence of human pituitary feedback. E2 increased DIO2 and OATP1C1 mRNA, showed the same directional pattern in representative protein blots, and increased the supernatant T3/T4 ratio; these changes were attenuated by ICI, supporting ER-sensitive estradiol responsiveness of candidate thyroid hormone transport and activation pathways.

The nominal E2 concentration selected for the Human Astrocytes intervention experiments (15,000 pg/mL) was chosen on the basis of dose-response and viability screening. Although this concentration was within the same order of magnitude as the third-trimester serum E2 levels observed in the clinical cohort, total circulating E2 in human serum and nominal E2 concentration in culture medium are not directly equivalent because protein binding, medium composition, and cellular exposure conditions differ substantially. The cell experiments should therefore be interpreted as mechanistic tests of E2 responsiveness in a controlled glial-like system rather than as a quantitative replication of late-pregnancy endocrine exposure.

Although pituitary folliculostellate cells and astrocytes share glial-like characteristics, the present study did not directly validate Human Astrocytes against human pituitary folliculostellate cells or compare the expression profiles of DIO2, OATP1C1, or estrogen receptors between these cell types (15, 24–31). Human Astrocytes were used as an accessible glial-like platform rather than as a direct model of human pituitary regulation. Accordingly, the cell findings should be interpreted as evidence of estradiol responsiveness of candidate thyroid hormone transport and activation pathways in this system, not as validation of folliculostellate-cell equivalence or a human pituitary mechanism.

From a clinical perspective, these data do not support changes in clinical interpretation, screening, or treatment recommendations in late pregnancy. Because maternal or fetal outcomes, screening strategies, and treatment responses were not assessed, whether combined interpretation of FT4 and TSH has any incremental prognostic or management value will require prospective studies using pregnancy-specific reference intervals and maternal–fetal outcome endpoints.

Several limitations should be considered. The clinical cohort was retrospective and single-center and did not include maternal or infant outcomes. Important determinants of thyroid physiology during pregnancy—including iodine status, iron status, body mass index, inflammatory activity, glucocorticoid-related factors (39), and medication exposure—were not uniformly available, and residual confounding may have affected both the direction and magnitude of the observed association between E2 and FT4. In addition, TT4, TT3, and TBG were not included in the reported clinical analyses, and FT4 was measured by routine immunoassay; therefore, binding-related and assay-related influences on the observed clinical phenotype could not be assessed (35, 36). The FT4 cutoff used to define IMH was based on routine hospital laboratory intervals rather than pregnancy-specific reference intervals derived from the study population; accordingly, some degree of misclassification in late pregnancy cannot be excluded. The two rat models captured complementary rather than identical aspects of the clinical phenotype, and pituitary T3/T4 values were obtained from an extraction-based protocol that provided comparative tissue readouts. The Human Astrocyte experiments provided evidence from a glial-like human cell system but did not reproduce the full pituitary microenvironment. Several experimental hormonal and gene-expression measures were based on relatively small datasets, and multiplicity was handled within individual analyses rather than through a single global correction across endpoints; these analyses should therefore be regarded as exploratory. Prospective studies incorporating pregnancy-specific reference intervals, maternal–fetal outcomes, and intervention assessment are needed to define the clinical significance of these findings.

## 5 Conclusion

**Figure 5:**
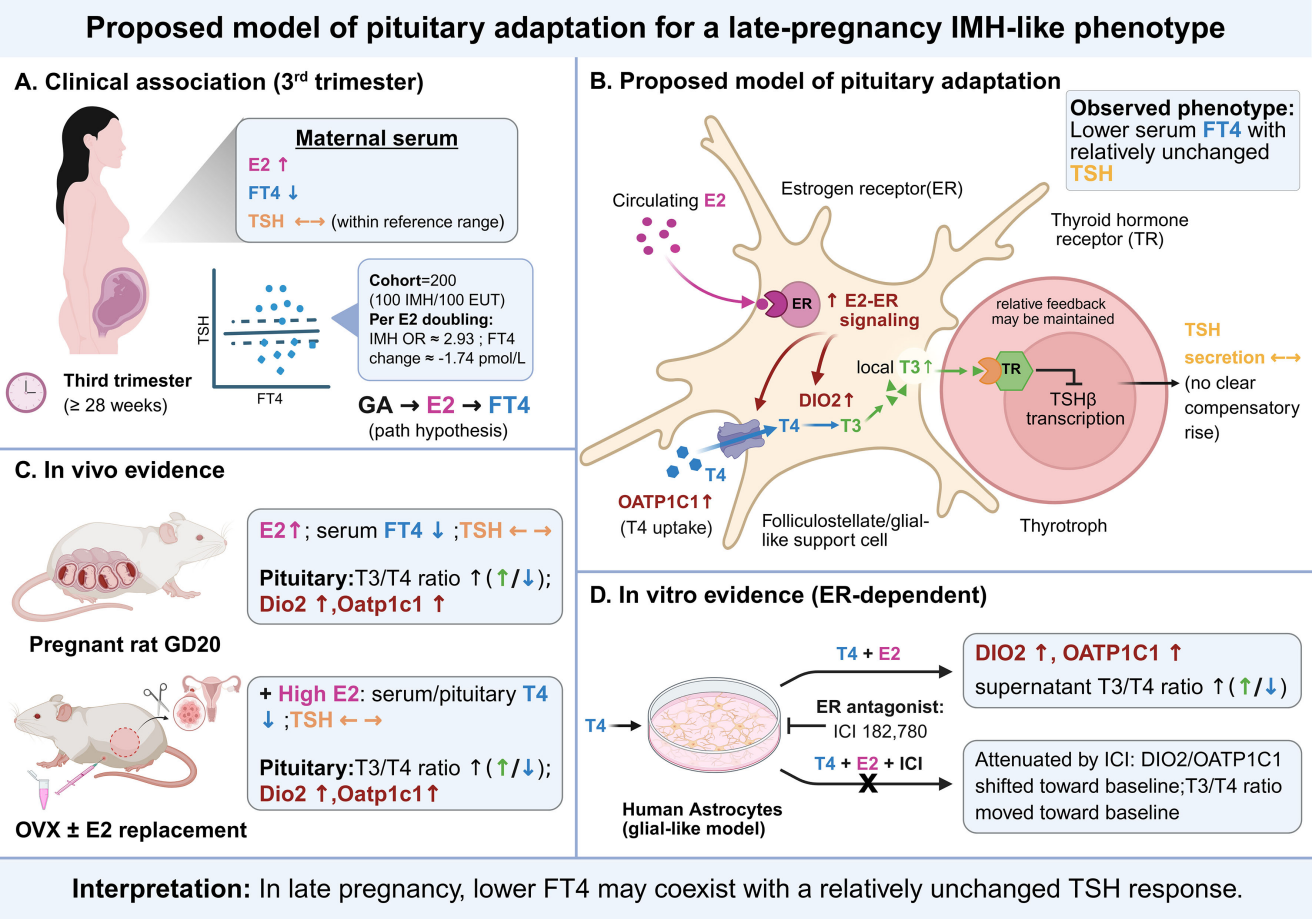
Schematic summary of a proposed model of pituitary adaptation for a late-pregnancy IMH-like phenotype. This schematic summarizes the direction of the observed changes and the proposed biological relationships derived from the present clinical, animal, and cell data. The proposed relationships were not directly assessed in the clinical cohort and should not be interpreted as evidence of a validated human pituitary mechanism. Arrows “↑” and “↓” indicate an increase/upregulation or decrease/downregulation, respectively. “↔” indicates no significant change or values remaining within the reference range. “→” indicates a directional pathway or transformation. “—|” and “X” indicate inhibition, antagonism, or blockade of a pathway. Color coding: magenta, E2; dark blue, FT4/T4; orange, TSH; green, T3. Dark red highlights the E2-activated cascade, including E2–ER signaling and downstream Dio2/DIO2 and Oatp1c1/OATP1C1-related processes. Dio2/DIO2, type II deiodinase; Dio3/DIO3, type III deiodinase; E2, estradiol; ER, estrogen receptor; EUT, euthyroid controls; FT4, free thyroxine; GA, gestational age; GD, gestational day; ICI, ICI 182,780; IMH, isolated maternal hypothyroxinemia; Oatp1c1/OATP1C1, organic anion transporting polypeptide 1c1; OR, odds ratio; OVX, ovariectomized; T3, triiodothyronine; T4, thyroxine; TR, thyroid hormone receptor; TSH, thyroid-stimulating hormone; TSHβ, thyroid-stimulating hormone beta subunit. Created using BioRender.com.

In summary, higher third-trimester E2 was associated with IMH and lower FT4 in the clinical cohort. Complementary rat experiments and a glial-like human astrocyte system showed changes compatible with altered local thyroid hormone handling under high-E2 conditions. Because pituitary thyroid hormone metabolism was not directly assessed in the clinical cohort, the proposed model of pituitary adaptation is not demonstrated in humans and should be regarded as exploratory and hypothesis-generating (**Figure 5**). Because the study did not assess maternal–fetal outcomes, screening performance, or treatment response, prospective studies using pregnancy-specific reference intervals and outcome-based endpoints are required before any clinical implications can be defined.

## Data Availability

The raw data supporting the conclusions of this article will be made available by the authors, without undue reservation.
