# Supplementary material for: Third-trimester estradiol is associated with isolated maternal hypothyroxinemia and attenuated TSH–FT4 coupling: evidence from rats and human astrocytes
Source: Front Endocrinol (Lausanne). 2026 Apr 30;17:1795855. doi: 10.3389/fendo.2026.1795855 (PMC13171308; doi:10.3389/fendo.2026.1795855)
Supplement: Supplementary file 1 [file DataSheet1.pdf]

Table S1. Sensitivity analysis and partial correlation analysis (n = 200).

Model and Variable	OR / r	95% CI	P value
**S1A. Sensitivity Analysis (IMH Status)**	**OR**	**95% CI**	
E2_resid [1]	2.925	1.136, 7.534	< 0.05
GA (wk)	1.034	0.881, 1.213	0.687
Age (yr)	1.025	0.949, 1.107	0.529
**S1B. Partial Correlation (ln(TSH)vs. FT4) [2]**	**r**		
Total population (n = 200)	0.012	--	0.865
EUT group (n = 100)	-0.033	--	0.749
IMH group (n = 100)	-0.064	--	0.533

Notes:

[1] E2_resid represents the residuals from log2(E2) regressed on GA and maternal age.

[2] Partial correlation coefficients (r) adjusted for GA and maternal age.

Abbreviations: CI, confidence interval; E2, estradiol; EUT, euthyroid controls; FT4, free thyroxine; GA, gestational age; IMH, isolated maternal hypothyroxinemia; ln, natural logarithm; log2, base-2 logarithm; n, number of subjects; OR, odds ratio; r, partial correlation coefficient; TSH, thyroid-stimulating hormone.

Table S2. Comparison of serum and pituitary thyroid hormone and E2 levels in rats across gestational stages.

Parameter (unit)	NP	GD6	GD12	GD20	Overall P value
**Serum**					
FT3 (pmol/L)	5.49 ± 0.54 (a)	5.41 ± 0.40 (a)	5.44 ± 0.32 (a)	4.66 ± 0.34 (b)	< 0.0001
FT4 (pmol/L)	24.33 ± 3.44 (a)	21.94 ± 3.14 (a)	20.82 ± 2.67 (a)	17.19 ± 3.61 (b)	< 0.0001
TSH (ng/mL)	1.32 ± 0.42 (a)	1.63 ± 0.56 (a)	1.50 ± 0.38 (a)	1.52 ± 0.54 (a)	0.5440
E2 (pg/mL)	25.61 ± 7.30 (a)	29.44 ± 7.02 (a)	34.86 ± 9.25 (a)	64.11 ± 16.89 (b)	< 0.0001
**Pituitary**					
T3 (pmol/L)	5.74 ± 0.55	5.49 ± 0.81	5.79 ± 0.62	5.59 ± 0.58	0.911
T4 (pmol/L)	67.00 ± 7.52 (a)	63.64 ± 8.07 (a)	60.26 ± 2.42 (ab)	50.95 ± 4.06 (b)	0.015
T3/T4 ratio	0.087 ± 0.014	0.088 ± 0.018	0.096 ± 0.013	0.111 ± 0.019	0.18
TSH (ng/mL)	18.61 ± 1.14	19.64 ± 1.76	18.68 ± 0.88	19.20 ± 0.78	0.763

Notes: Data are presented as mean ± SD. Serum sample sizes: FT3, FT4, and E2, n = 12; TSH, n = 10. Pituitary sample sizes: T3, T4, and TSH, n = 4; T3/T4 ratio, n = 4. n denotes independent

biological replicates. Statistical differences were assessed by one-way ANOVA or Welch ANOVA, followed by post-hoc multiple comparison tests. Within each row, means followed by different superscript letters (a, b) indicate a statistically significant difference ($P < 0.05$).

Abbreviations: ANOVA, analysis of variance; E2, estradiol; FT3, free triiodothyronine; FT4, free thyroxine; GD, gestational day; n, number of subjects; NP, non-pregnant; SD, standard deviation; T3, triiodothyronine; T4, thyroxine; TSH, thyroid-stimulating hormone.

Table S3. Pituitary mRNA expression of thyroid-related genes across different gestational stages in rats.

**Gene Symbol**	**F-value (df = 3, 12)**	**Overall P value**	**Significant Pairwise Comparisons (Post-hoc) [1]**
Tshb	0.027	0.9936	ns
Dio2	9.557	< 0.01	GD20 > NP (P < 0.01); GD20 > GD6 (P < 0.01)
Dio3	3.019	0.0717	ns
Mct8	0.361	0.7824	ns
Oatp1c1	4.739	< 0.05	GD20 > NP (P < 0.05); GD20 > GD6 (P < 0.05)

Notes: n = 4 per group. n denotes independent biological replicates. Relative mRNA expression was determined using the $2^{-\Delta\Delta Ct}$ method and normalized to the NP group. Overall P values were determined by one-way ANOVA.

[1] Pairwise comparisons were performed using Tukey's post-hoc test.

"ns" indicates no statistically significant difference (adjusted $P \geqslant 0.05$) in any pairwise comparisons.

Abbreviations: ANOVA, analysis of variance; df, degrees of freedom; Dio2, type II deiodinase; Dio3, type III deiodinase; F, F-statistic; GD, gestational day; Mct8, monocarboxylate transporter 8; mRNA, messenger RNA; NP, non-pregnant; ns, not significant; Oatp1c1, organic anion transporting polypeptide 1c1; Tshβ, thyrotropin subunit beta.

Table S4. Exploratory GLM analysis of factors associated with ln(TSH) levels (n = 40).

**Predictor**	**F (df)**	**P value**	**Partial eta2**	**B (unstandardized)**	**95% CI for B**
**Dependent variable: ln(TSH)[1]**					
Corrected Model	0.96 (5, 34)	0.455	0.12	--	--
GA Group	0.63 (3, 34)	0.603	0.05	--	--
FT4	1.05 (1, 34)	0.312	0.03	-0.018	-0.054, 0.018
log2(E2)	0.40 (1, 34)	0.533	0.01	0.089	-0.198, 0.377

Notes:

[1] This exploratory model included GA group, FT4, and log2(E2) as simultaneous predictors of ln(TSH). Model fit: R^2 = 0.124, Adjusted R^2 = -0.005. No statistically significant independent associations were detected in this exploratory model.

Abbreviations: B, unstandardized coefficient; CI, confidence interval; df, degrees of freedom; E2, estradiol; eta^2, partial eta squared; F, F-statistic; FT4, free thyroxine; GA, gestational age; GLM, General Linear Model; ln, natural logarithm; log2, base-2 logarithm; n, number of subjects; R^2, coefficient of determination; TSH, thyroid-stimulating hormone.

Table S5. Spearman correlation analysis of thyroid-estradiol axis parameters in total and stratified rat samples.

Analysis Group	Variable Pair	rho (Spearman's ρ)	P value
**A. Total Sample (n = 40)**			
	FT4 vs. log2(E2)	-0.65	< 0.001
	ln(TSH)vs. FT4	-0.29	0.08
	ln(TSH)vs. log2(E2)	0.15	0.36
**B. Stratified Analysis (n = 10 per group)**			
NP	FT4 vs. log2(E2)	-0.22	0.53
	ln(TSH)vs. FT4	-0.39	0.26
	ln(TSH)vs. log2(E2)	0.15	0.68
GD6	FT4 vs. log2(E2)	-0.24	0.5
	ln(TSH)vs. FT4	-0.21	0.56
	ln(TSH)vs. log2(E2)	-0.08	0.83
GD12	FT4 vs. log2(E2)	-0.685	< 0.05
	ln(TSH)vs. FT4	-0.38	0.28
	ln(TSH)vs. log2(E2)	0.03	0.93
GD20	FT4 vs. log2(E2)	-0.49	0.15
	ln(TSH)vs. FT4	-0.25	0.49
	ln(TSH)vs. log2(E2)	0.28	0.44

Notes: Analysis was performed using Spearman's rank correlation. For consistency across variable pairs involving ln(TSH), all correlations in this table were performed in the subset with

complete FT4, E2, and TSH data (n = 40 total; n = 10 per gestational group). By contrast, the FT4 versus log2(E2) association shown in Figure 2D and Table 4A used all rats with available FT4 and E2 measurements (n = 48). Results from the stratified analysis (Panel B) are considered exploratory due to the small sample size (n = 10 per group). n denotes independent biological replicates.

Abbreviations: E2, estradiol; FT4, free thyroxine; GD, gestational day; ln, natural logarithm; log2, base-2 logarithm; n, number of subjects; NP, non-pregnant; rho, Spearman's rank correlation coefficient; TSH, thyroid-stimulating hormone.

Table S6. Effects of E2 replacement on serum and pituitary thyroid hormone profiles in OVX rats.

Parameter (unit)	OVX	Sham	OVX+Veh	OVX+E2 (0.7)	OVX+E2 (2.1)	Overall P value
**Model Validation (n=9)**						
Serum E2 (pg/mL)	11.79 ± 4.05 (a)	28.15 ± 3.46 (b)	12.60 ± 3.06 (a)	46.77 ± 6.72 (c)	70.25 ± 9.91 (d)	< 0.0001 [1]
**Serum Thyroid Function (n=9)**						
FT3 (pmol/L)	5.35 ± 0.79	5.59 ± 0.56	5.37 ± 0.61	5.65 ± 0.52	5.81 ± 0.48	0.442
FT4 (pmol/L)	22.20 ± 3.15 (a)	22.80 ± 3.66 (a)	21.69 ± 3.03 (a)	19.95 ± 1.75 (ab)	16.52 ± 2.38 (b)	< 0.001
TSH (ng/mL)	1.71 ± 0.44	1.58 ± 0.45	1.66 ± 0.46	1.62 ± 0.39	1.60 ± 0.40	0.97
**Pituitary Thyroid Status (n=3)**						
Pituitary T3 (pmol/L)	5.24 ± 0.52	5.47 ± 0.45	5.20 ± 0.38	5.52 ± 0.48	5.74 ± 0.80	0.737
Pituitary T4 (pmol/L)	59.98 ± 5.72 (a)	61.16 ± 5.39 (a)	58.90 ± 3.43 (a)	52.94 ± 2.23 (ab)	45.25 ± 4.80 (b)	0.008
Pituitary T3/T4 ratio	0.088 ± 0.016 (a)	0.090 ± 0.007 (a)	0.088 ± 0.001 (a)	0.104 ± 0.007 (ab)	0.127 ± 0.004 (b)	0.001

Pituitary TSH (ng/mL)	20.59 ± 1.74	19.96 ± 0.85	20.37 ± 0.94	19.71 ± 0.78	19.79 ± 1.08	0.846

Notes: Data are presented as mean ± SD. n denotes independent biological replicates. Dose of E2 is expressed as μg/100 g body weight.

[1] For serum E2, comparison was performed using Welch ANOVA. For other parameters, one-way ANOVA was applied.

Within each row, means followed by different superscript letters (a, b, c, d) indicate a statistically significant difference ($P < 0.05$) based on post-hoc tests.

Abbreviations: ANOVA, analysis of variance; E2, estradiol; FT3, free triiodothyronine; FT4, free thyroxine; n, number of subjects; OVX, ovariectomized; SD, standard deviation; Sham, sham-operated; T3, triiodothyronine; T4, thyroxine; TSH, thyroid-stimulating hormone; Veh, vehicle control.

Table S7. Summary of pituitary mRNA expression differences among groups in the OVX rat model.

Gene Symbol	F-value (df = 4, 10)	Overall P value	Significant Pairwise Comparisons (Post-hoc) [1]
Tshb	1.848	0.196	ns
Dio2	7.778	< 0.01	OVX+E2(2.1) > OVX (P < 0.01); OVX+E2(2.1) > OVX+Veh (P < 0.01)
Oatp1c1	28.17	< 0.0001	OVX+E2(2.1) > OVX (P < 0.0001); OVX+E2(2.1) > Sham (P < 0.001); OVX+E2(2.1) > OVX+Veh (P < 0.0001); OVX+E2(2.1) > OVX+E2(0.7) (P < 0.001)
Mct8	1.894	0.188	ns
Dio3	2.73	0.09	ns

Notes: n = 3 per group. n denotes independent biological replicates. Overall P values were determined by one-way ANOVA.

[1] Pairwise comparisons were performed using Tukey's post-hoc test.

"ns" indicates no statistically significant difference (adjusted P ⩾ 0.05).

Abbreviations: ANOVA, analysis of variance; df, degrees of freedom; Dio2, type II deiodinase; Dio3, type III deiodinase; E2, estradiol; F, F-statistic; Mct8, monocarboxylate transporter 8;

mRNA, messenger RNA; ns, not significant; Oatp1c1, organic anion transporting polypeptide 1c1; OVX, ovariectomized; Sham, sham-operated; Tshβ, thyrotropin subunit beta; Veh, vehicle control.

Table S8. Statistical comparisons of mRNA expression in human astrocytes treated with varying concentrations of E2.

**Comparison (pg/mL)**	**DIO2**	**DIO3**	**MCT8**	**OATP1C1**
Overall P (ANOVA)	< 0.01	0.141	< 0.001	< 0.001
0 vs. 50	0.999	0.253	0.999	> 0.999
0 vs. 1000	0.515	0.997	0.411	0.201
0 vs. 2500	0.198	0.814	0.34	< 0.01
0 vs. 15000	< 0.01	> 0.999	< 0.001	< 0.001
0 vs. 50000	0.845	0.535	0.11	0.463
50 vs. 1000	0.727	0.455	0.551	0.221
50 vs. 2500	0.335	0.871	0.469	< 0.05
50 vs. 15000	< 0.01	0.179	< 0.001	< 0.001
50 vs. 50000	0.964	0.989	0.166	0.498
1000 vs. 2500	0.974	0.966	> 0.999	0.482
1000 vs. 15000	< 0.05	0.981	< 0.01	< 0.001
1000 vs. 50000	0.988	0.787	0.936	0.987
2500 vs. 15000	0.15	0.691	< 0.01	< 0.01
2500 vs. 50000	0.76	0.995	0.969	0.211
15000 vs. 50000	< 0.05	0.41	< 0.01	< 0.001

Notes: n = 3 independent experiments per group. Data were analyzed by one-way ANOVA to determine overall significance. Pairwise comparisons were performed using Tukey's post-hoc

test to calculate adjusted P values.

Abbreviations: ANOVA, analysis of variance; DIO2, type II deiodinase; DIO3, type III deiodinase; E2, estradiol; MCT8, monocarboxylate transporter 8; n, number of independent experiments; OATP1C1, organic anion transporting polypeptide 1c1.

Table S9. Impact of E2 and estrogen receptor inhibition on the transcription of key thyroid-related genes in human astrocytes.

**Comparison**	**DIO2**	**OATP1C1**
Overall P (ANOVA)	< 0.01	< 0.01
T4 vs. T4+E2	< 0.05	< 0.01
T4 vs. T4+ICI	0.997	0.998
T4 vs. T4+E2+ICI	0.968	0.986
T4+E2 vs. T4+ICI	< 0.05	< 0.05
T4+E2 vs. T4+E2+ICI	< 0.05	< 0.05
T4+ICI vs. T4+E2+ICI	0.914	0.999

Notes: n = 3 independent experiments per group. Overall significance was determined by one-way ANOVA. Pairwise comparisons were performed using Tukey's post-hoc test to calculate adjusted P values.

Abbreviations: ANOVA, analysis of variance; DIO2, type II deiodinase; E2, estradiol; ICI, ICI 182,780 (estrogen receptor inhibitor); mRNA, messenger RNA; n, number of independent experiments; OATP1C1, organic anion transporting polypeptide 1c1; T4, thyroxine.

Table S10. Primer sequences used for Real-Time Quantitative PCR

**Target Gene**	**Gene Symbol**	**Species**	**Primer Type**	**Sequence (5' to 3')**
Glyceraldehyde-3-phosphate dehydrogenase	GAPDH	Human	Forward	ACAACTTTGGTATCGTGGAAGG
			Reverse	GCCATCACGCCACAGTTTC
	Gapdh	Rat	Forward	AAGCCCATCACCATCTTCCA
			Reverse	ATGGCATGGACTGTGGTCAT
Iodothyronine deiodinase 2	DIO2	Human	Forward	GGATGCCCCCAATTCCAGTG
			Reverse	GGCTCGTGAAAGGAGGTCAAG
	Dio2	Rat	Forward	AATTATGCCTCGGAGAAGACCG
			Reverse	GGCAGTTGCCTAGTGAAAGGT
Iodothyronine deiodinase 3	DIO3	Human	Forward	ATCCTCGACTACGCGCAAG
			Reverse	GGGATGATGTAGGGAGAGTCC
	Dio3	Rat	Forward	CTCGACTACGCACAAGGGAC
			Reverse	GGTGGGCTTCCTCGATGTA
Monocarboxylate transporter 8	MCT8	Human	Forward	CCACGCCTACGGTAGAGAC
			Reverse	CAGAGTTATGGATGCCGAAGATG
	Mct8	Rat	Forward	CGGCTGGATAGTGGTGTTTG
			Reverse	CAGAGTTATGGATGCCGAAGATG
Organic anion transporting polypeptide 1c1	OATP1C1	Human	Forward	GGAGTTGGAACACTGCTCATT
			Reverse	CTTGACTCTAGGAGACACGGA
	Oatp1c1	Rat	Forward	AAATCGGGAATCTCCTGGTCA

			Reverse	AGCAATGAGCATAGTTCCGAAG
Thyrotropin subunit beta	Tshβ	Rat	Forward	CCACCATCTGTGCTGGGTATTG
			Reverse	GCATCCTGGTATTTCCACCGTTC

Table S11. Primary antibodies used for Western blot.

Target Protein	Dilution	Manufacturer	Catalog Number
DIO2	1:1000	Abcam	ab77779
DIO3	1:500	Invitrogen	PA5-26537
MCT8	1:500	Abcam	ab192828
OATP1C1	1:500	Invitrogen	PA5-115919
β-ACTIN	1:7000	Abways	AB0035
